# Smoking, environmental tobacco smoke, and risk of renal cell cancer: a population-based case-control study

**DOI:** 10.1186/1471-2407-8-387

**Published:** 2008-12-24

**Authors:** Ryan P Theis, Suzanne M Dolwick Grieb, Deborah Burr, Tariq Siddiqui, Nabih R Asal

**Affiliations:** 1 Department of Epidemiology and Health Policy Research, College of Medicine, University of Florida, Florida, USA; 2 Department of Epidemiology and Biostatistics, College of Public Health and Health Professions, University of Florida, Florida, USA; 3 Hematology and Oncology Division, Department of Medicine, College of Medicine, University of Florida, Florida, USA

## Abstract

**Background:**

Kidney and renal pelvis cancers account for 4% of all new cancer cases in the United States, among which 85% are renal cell carcinomas (RCC). While cigarette smoking is an established risk factor for RCC, little is known about the contribution of environmental tobacco smoke (ETS) to RCC incidence. This study assesses the role of smoking and ETS on RCC incidence using a population-based case-control design in Florida and Georgia.

**Methods:**

Incident cases (n = 335) were identified from hospital records and the Florida cancer registry, and population controls (n = 337) frequency-matched by age (+/- 5 years), gender, and race were identified through random-digit dialing. In-person interviews assessed smoking history and lifetime exposure to ETS at home, work, and public spaces. Home ETS was measured in both years and hours of exposure. Odds ratios and 95% confidence intervals were calculated using logistic regression, controlled for age, gender, race, and BMI.

**Results:**

Cases were more likely to have smoked 20 or more pack-years, compared with never-smokers (OR: 1.35, 95% CI: 0.93 – 1.95). A protective effect was found for smoking cessation, beginning with 11–20 years of cessation (OR: 0.39, 95% CI: 0.18–0.85) and ending with 51 or more years of cessation (OR: 0.11, 95% CI: 0.03–0.39) in comparison with those having quit for 1–10 years. Among never-smokers, cases were more likely to report home ETS exposure of greater than 20 years, compared with those never exposed to home ETS (OR: 2.18; 95% CI: 1.14–4.18). Home ETS associations were comparable when measured in lifetime hours of exposure, with cases more likely to report 30,000 or more hours of home ETS exposure (OR: 2.37; 95% CI: 1.20–4.69). Highest quartiles of combined home/work ETS exposure among never-smokers, especially with public ETS exposure, increased RCC risk by 2 to 4 times.

**Conclusion:**

These findings confirm known associations between smoking and RCC and establish a potential etiologic role for ETS, particularly in the home. Differences in methods of retrospective measurement of lifetime smoking and ETS exposure may contribute to discrepancies in measures of associations across studies, and should be addressed in future research.

## Background

Kidney and renal pelvis cancers account for nearly 4% of all new cancer cases in the United States, with 54,390 new cases estimated for the year 2008 [1,2]. Incidence rates have almost doubled over the past 30 years – from 7.1 per 100,000 in 1975 to 13.4 per 100,000 in 2005 [1]. Most of these cases are renal cell carcinomas (RCC), accounting for approximately 85% of all renal tumors [3]. Known genetic predispositions explain 2% of RCC cases [3], suggesting that increases in incidence are due largely to environmental factors.

The association between RCC and cigarette smoking is well established, although reported risk increases for ever-smokers compared with never-smokers are moderate. In a recent meta-analysis, ever-smoking produced a relative risk for RCC of 1.38, and risk increases were generally greater among men (RR = 1.50) than women (RR = 1.27) [4]. Despite these modest risk increases, dose-response associations and cessation effects have been consistently reported. The International Agency for Research on Cancer has concluded that sufficient evidence exists for a causal association between cigarette smoking and RCC [5].

Few studies have explored the potential associations between environmental tobacco smoke (ETS) and RCC [6,7]. The Surgeon General's most recent report on involuntary tobacco smoke concluded that living with a smoker increases lung cancer risk by 20 to 30%, and that ETS may also contribute to cancers of the breast and nasal sinus cavity [8]. A case-control study of passive smoking and overall cancer risk found that individuals ever married to smokers were 1.6 times more likely than those never married to smokers to develop cancer at any site [9].

The present paper reports findings from a population-based case-control study of RCC in Florida and Georgia, evaluating the role of tobacco smoke exposure for cigarettes and ETS – including duration of ETS exposure in the home, workplace, and in other public or private locations.

## Methods

Incident, histologically confirmed cases of RCC were identified from hospital records in North Florida and through the Florida Cancer Data System registry. All white and African-American cases aged 20 years or older and diagnosed between January 1, 2000 and December 31, 2004 were considered for inclusion. Cases were excluded if their cancer was diagnosed in a transplant kidney, if they were diagnosed with transitional cell tumors, or if they did not reside in Florida or Georgia. Among 417 living, eligible cases contacted, 304 (73%) participated in the study. Fifteen additional cases were included from urology clinics in North-Central Florida, four through Emory University Hospital in Atlanta, Georgia, and 12 through the Malcom Randall VA Medical Center in Gainesville, Florida, producing a total sample of 335 cases. The resulting sample was drawn from 22 counties in North-Central Florida, two counties in South Florida, two counties in the Florida Panhandle, two counties in Southeastern Georgia, and Atlanta.

A sample of population controls was concurrently identified using random-digit dialing (RDD) [10], frequency-matched to cases by age (+/- 5 years), gender and race. Sampling frames were based on permutations of the telephone numbers of cases, holding area code and three-digit prefix constant, which allowed controls to be identified from the same cities as cases. During the first year of the study, frequencies from Surveillance, Epidemiology, and End Results (SEER) data [11] were used for matching until an adequate case sample was acquired. Respondents were eligible as controls if they met matching criteria and reported no history of kidney cancer. Among 801 eligible respondents contacted by telephone, 337 (42%) participated as controls.

Ethical approval was obtained from the institutional review boards of the University of Florida (UF IRB-01) and secondary study sites. Subjects who provided informed consent to participate were interviewed in person between August 2003 and December 2006 using a structured questionnaire, minimizing the potential for interviewer bias [12]. The questionnaire assessed medical, occupational, and family histories, lifetime history of use of medications, tobacco, coffee, tea, and artificial sweeteners, and lifetime exposure to radiation, pesticides, and environmental tobacco smoke. Dietary information was collected using a Block food frequency questionnaire [13]. Body mass index (BMI) was calculated at time of interview by measuring height in centimeters and weight in kilograms (*kg/m*^2^).

Subjects were classified as "ever-smokers" of cigarettes if they reported smoking at least 100 cigarettes during their lifetime. Subjects were asked to indicate the number of cigarettes they smoked per day separately for each of eight age-decade groups. Discrete values from each group were summed to produce a lifetime exposure value, using validated methods for the retrospective calculation of pack-years (1 pack-year = 1 pack/day for one year) [14] and taking into account periods of temporary cessation. Ever-smokers were asked whether they normally inhaled into the chest or into the mouth when they smoked.

Exposure to ETS was assessed separately for home, workplace, and public or private locations. For home exposure, subjects were asked whether they had ever lived with someone who smoked inside their home for at least one year. For each home indicated, years of co-habitation and average number of hours per day of ETS exposure were collected. Summary measures of home ETS exposure duration and lifetime hours of exposure (*years *× *hours*) were calculated by adding estimates for each home.

For workplace ETS exposure, subjects were asked whether they had been exposed to others' cigarette smoke in the workplace for at least one year, the number of years of employment in such jobs, and the average number of hours per day exposed. Additionally, subjects were asked to indicate the average number of smokers within 10 feet of them in the workplace.

Exposure to ETS in "cars, public transport, restaurants, bars, or other public/private places" was also evaluated (*public ETS exposure*). Subjects were considered exposed if they reported exposure in these places for at least one hour per week, during the time in their life when they frequented such locations "most often". Frequency of exposure was collected in hours per week and grouped into three categories: (1) 1–2 hours; (2) 3–6 hours; or (3) 7 or more hours. Because public ETS is common in bars and restaurants, analyses were controlled for weekly alcohol consumption in the past year. This variable was constructed from responses to questions on beer, wine, and liquor, with one point assigned for each beverage the subject reported drinking at least once per week (score range: 0 – 3). An additional point was added for subjects reporting to have consumed more alcohol in the past than presently.

Statistical analyses were performed using SPSS 14.0 software (SPSS Inc., Chicago). Comparisons of demographic factors employed the Pearson χ^2 ^test for independence. Relative risk statistics were estimated by the odds ratio (OR) and 95% confidence interval (CI), using unconditional logistic regression. Adjusted models controlled for age, gender, race, smoking, and BMI. Tests for trend employed the Wald χ^2 ^statistic, computed for continuous variables in adjusted models. Analyses of ETS exposure were performed for never-smokers only.

## Results

Cases and controls did not differ significantly with respect to gender, race, or household income (Table 1). The difference in mean ages between cases (66 years) and controls (62 years) was statistically significant (p = 0.001). Cases were interviewed between 0.4 years and 6.3 years following diagnosis, with a mean follow-up of 3.1 years. Compared with healthy BMI (18.5 – 24.9), being obese (BMI 30 – 39) was associated with an 80% increase in RCC risk for both men (OR = 1.8; 95% CI: 0.9 – 3.6) and women (OR = 1.8; 95% CI: 1.0 – 3.3). Based on these findings, all multivariate analyses were controlled for BMI, in addition to age, gender and race. Hypertension is also considered an important risk factor in the epidemiology of RCC [3], although no associations were found in the present study – an effect that was likely due to misclassification of subjects who claimed to be hypertensive by self-report, but were never formally diagnosed. Hypertension was therefore not included as a covariate in subsequent analyses.

**Table 1:** Demographic characteristics of RCC cases and controls, Florida and Georgia

	**Cases (%)**	**Controls (%)**	**Pearson χ^2^**	**p-value**
**Age at interview**			17.239	0.008
20 – 29 years	1 (0.3%)	9 (2.7%)		
30 – 39 years	2 (0.6%)	11 (3.3%)		
40 – 49 years	25 (7.5%)	38 (11.3%)		
50 – 59 years	63 (18.8%)	64 (19.0%)		
60 – 69 years	108 (32.2%)	98 (29.1%)		
70 – 79 years	95 (28.4%)	81 (24.0%)		
80 + years	41 (12.2%)	36 (10.7%)		

**Gender**			0.220	0.644
Female	154 (46.0%)	161 (47.8%)		
Male	181 (54.0%)	176 (52.2%)		

**Race**			0.052	0.851
White	262 (78.2%)	266 (78.9%)		
African-American	73 (21.8%)	71 (21.1%)		

**Education**			3.668	0.160
Less than high school	41 (12.3%)	28 (8.3%)		
High school diploma	184 (55.1%)	183 (54.3%)		
Bachelor's or higher	109 (32.6%)	126 (37.4%)		

**Annual household income**			8.998	0.342
Less than $10,000	19 (5.9%)	25 (7.5%)		
$10,000 – $14,999	19 (5.9%)	20 (6.0%)		
$15,000 – $19,999	19 (5.9%)	28 (8.4%)		
$20,000 – $24,999	41 (12.7%)	24 (7.2%)		
$25,000 – $34,999	48 (14.8%)	44 (13.3%)		
$35,000 – $49,999	59 (18.2%)	54 (16.3%)		
$50,000 – $74,999	58 (17.9%)	65 (19.6%)		
$75,000 and higher	53 (16.4%)	65 (19.6%)		
Not reported	8 (2.5%)	7 (2.1%)		

**Body mass index**			6.384	0.094
18.5 – 24.9	50 (15.0%)	70 (21.3%)		
25.0 – 29.9	118 (35.3%)	123 (37.4%)		
30.0 – 39.9	135 (40.4%)	111 (33.7%)		
40.0 +	31 (9.3%)	25 (7.6%)		

A multivariate logistic regression model was used to calculate odds ratios for lifetime smoking measured in years, pack-years, inhalation and smoking cessation (Table 2). RCC risk increased with increasing duration of smoking in years (p = 0.055), although few odds ratios across decade-intervals were significant. When compared with those smoking for less than 20 years, those who smoked for 20 years or longer experienced a 60% increase in RCC risk. Trends between RCC and direct smoking were stronger when exposure was measured in pack-years (p = 0.014). Those who smoked 20 or more pack-years experienced a marginally significant 30% risk increase compared with never-smokers. This association was greater when smokers of 20 or more pack-years were compared with those smoking less than 20 pack-years.

**Table 2:** Crude and adjusted odds ratios for renal cell carcinoma: cigarettes and cigarette smoking cessation

	**Cases** **(%)**	**Controls** **(%)**	**OR** **(95% CI)**	**AOR** **(95% CI)^a^**	**χ^2^** **trend^b^**
**Lifetime cigarette smoking**					
					
**Duration (years)**					p = 0.055
Never-smokers	131 (39%)	134 (40%)	1.00 (ref.)	1.00 (ref.)	
1 – 10 years	22 (7%)	46 (14%)	0.49 (0.28 – 0.86)	0.52 (0.29 – 0.93)	
11 – 20 years	29 (9%)	39 (12%)	0.76 (0.44 – 1.30)	0.73 (0.42 – 1.28)	
21 – 30 years	42 (13%)	39 (12%)	1.10 (0.67 – 1.81)	1.19 (0.71 – 1.99)	
31 – 40 years	56 (17%)	30 (9%)	1.91 (1.15 – 3.16)	1.87 (1.12 – 3.13)	
41 – 50 years	32 (10%)	29 (9%)	1.13 (0.65 – 1.97)	1.05 (0.60 – 1.84)	
51 + years	23 (7%)	20 (6%)	1.18 (0.62 – 2.24)	1.05 (0.54 – 2.04)	
Total	335 (100%)	337 (100%)			
					
**Dichotomous measure**					
< 20 years	176 (52%)	216 (64%)	1.00 (ref.)	1.00 (ref.)	
20 + years	159 (48%)	121 (36%)	1.60 (1.17 – 2.19)	1.57 (1.15 – 2.15)	
					
**Dose + duration (pack-years)**					p = 0.014
Never-smokers	131 (39%)	134 (40%)	1.00 (ref.)	1.00 (ref.)	
< 5 pack-years	29 (9%)	50 (15%)	0.64 (0.38 – 1.06)	0.68 (0.40 – 1.15)	
5 to < 10 pack-years	14 (4%)	24 (7%)	0.61 (0.30 – 1.22)	0.62 (0.31 – 1.27)	
10 to < 20 pack-years	33 (10%)	33 (10%)	1.04 (0.61 – 1.78)	1.11 (0.64 – 1.92)	
20 or more pack-years	128 (38%)	96 (29%)	1.39 (0.97 – 1.98)	1.35 (0.93 – 1.95)	
Total	335 (100%)	337 (100%)			
					
**Dichotomous measure**					
< 20 pack-years	207 (62%)	241 (71%)	1.00 (ref.)	1.00 (ref.)	
20 or more pack-years	128 (38%)	96 (29%)	1.53 (1.10 – 2.14)	1.48 (1.06 – 2.07)	
					
**Smoking technique^c^**					
Inhalation into mouth/throat only	29 (12%)	41 (19%)	1.00 (ref.)	1.00 (ref.)	
Inhalation into chest/lungs	176 (88%)	161 (81%)	1.89 (1.07 – 3.35)	1.83 (1.03 – 3.26)	-
Total	205 (100%)	202 (100%)			
					
**Cigarette cessation (years)^d^**					p < 0.001
1 – 10 years	48 (29%)	20 (14%)	1.00 (ref.)	1.00 (ref.)	
11 – 20 years	36 (22%)	27 (19%)	0.56 (0.27 – 1.14)	0.39 (0.18 – 0.85)	
21 – 30 years	28 (17%)	34 (24%)	0.34 (0.17 – 0.71)	0.24 (0.11 – 0.52)	
31 – 40 years	28 (17%)	30 (21%)	0.39 (0.19 – 0.81)	0.28 (0.13 – 0.61)	
41 – 50 years	18 (11%)	18 (13%)	0.42 (0.18 – 0.96)	0.23 (0.09 – 0.58)	
51 or more years	7 (4%)	11 (8%)	0.27 (0.09 – 0.78)	0.11 (0.03 – 0.39)	
Total	165 (100%)	140 (100%)			

^a ^Adjusted for age, gender, race and BMI. ^b ^Reported only for continuous variables in adjusted models.

^c ^Ever-smokers only. ^d ^Ex-smokers only.

Among ever-smokers, those who reported inhaling experienced an 83% increase in risk, compared with those who did not inhale. However, this association was attenuated when pack-years were included in the model (OR = 1.59; 95% CI: 0.88 – 2.86), suggesting confounding by dose. Among those who smoked 30 or more pack-years, 90% reported inhaling, compared with 73% of those who smoked less than 10 pack-years.

The strongest associations in this study were found for smoking cessation. Among ex-smokers, a trend of decreasing risk was observed across 10-year cessation intervals, using as a reference group those who had quit 1–10 years before interview. A 60% decrease in risk was observed for those who had quit 11–20 years prior to interview.

Table 3 shows results for ETS exposure among never-smokers. Exposure to home ETS significantly increased risk for RCC, whether tested as years (p = 0.010) or lifetime hours of exposure (p = 0.008). Compared with those reporting no home ETS exposure, those with greater than 20 years of exposure were more than twice as likely to develop RCC. Likewise, those with 30,000 hours or more of lifetime exposure – a figure equivalent to 5 hours a day for over 16 years – were 2.4 times more likely to develop RCC. Those exposed to ETS in the workplace for 1 – 20 years were twice as likely to develop RCC as those never exposed. However, this represents the middle range of workplace ETS exposure; at the higher range (greater than 20 years), no association was observed. No significant trends were otherwise found between workplace or public ETS exposure and RCC.

**Table 3:** Crude and adjusted odds ratios for renal cell carcinoma: environmental tobacco smoke exposure among never-smokers

	**Cases** **(%)**	**Controls** **(%)**	**OR** **(95% CI)**	**AOR** **(95% CI)^a^**	**χ^2 ^trend^b^**
**Home ETS exposure**					
Duration (years)					p = 0.010
No exposure	35 (27%)	43 (32%)	1.00 (ref.)	1.00 (ref.)	
1 – 20 years	38 (30%)	59 (44%)	0.79 (0.43 – 1.45)	0.86 (0.46 – 1.60)	
> 20 years	56 (43%)	32 (24%)	2.15 (1.15 – 4.01)	2.18 (1.14 – 4.18)	
Total	129 (100%)	134 (100%)			
					
Lifetime hours					p = 0.008
No exposure	37 (29%)	44 (33%)	1.00 (ref.)	1.00 (ref.)	
1 – 29,999 hours	41 (32%)	62 (46%)	0.79 (0.44 – 1.42)	0.83 (0.45 – 1.52)	
30,000 + hours	50 (39%)	28 (21%)	2.12 (1.12 – 4.01)	2.37 (1.20 – 4.69)	
Total	128 (100%)	134 (100%)			
					
**Workplace ETS exposure**					p = 0.740
Duration (years)					
No exposure	55 (43%)	73 (55%)	1.00 (ref.)	1.00 (ref.)	
1 – 20 years	48 (37%)	32 (24%)	1.99 (1.13 – 3.51)	2.09 (1.17 – 3.75)	
> 20 years	26 (20%)	29 (22%)	1.19 (0.63 – 2.25)	1.04 (0.54 – 2.01)	
Total	129 (100%)	134 (100%)			
					
Lifetime hours					p = 0.972
No exposure	55 (43%)	73 (55%)	1.00 (ref.)	1.00 (ref.)	
1 – 16,999 hours	38 (30%)	28 (21%)	1.80 (0.99 – 3.28)	1.83 (0.99 – 3.37)	
17,000 + hours	36 (28%)	33 (25%)	1.45 (0.80 – 2.61)	1.36 (0.74 – 2.49)	
Total	129 (100%)	134 (100%)			
					
Smoker proximity					p = 0.370
< 5 smokers within 10'^c^	44 (60%)	43 (71%)	1.00 (ref.)	1.00 (ref.)	
≥ 5 smokers within 10'	29 (40%)	18 (30%)	1.57 (0.76 – 3.24)	1.47 (0.70 – 3.08)	
Total	73 (100%)	61 (100%)			
					
**Public/private ETS exposure^d^**					-
< 1 hour per week	42 (33%)	54 (40%)	1.00 (ref.)	1.00 (ref.)	
1 – 2 hours per week	52 (41%)	46 (34%)	1.45 (0.83 – 2.56)	1.59 (0.88 – 2.87)	
3 – 6 hours per week	22 (17%)	16 (12%)	1.77 (0.83 – 3.78)	2.25 (0.99 – 5.09)	
≥ 7 hours per week	12 (9%)	18 (13%)	0.86 (0.37 – 1.97)	0.87 (0.37 – 2.05)	
Total	128 (100%)	134 (100%)			

^a ^Adjusted for age, gender, race, and BMI. ^b ^Reported only for continuous variables in adjusted models.

^c ^Assessed only for those reporting workplace ETS exposure. ^d ^Adjusted for age, gender, race, BMI, and weekly alcohol consumption.

Table 4 presents results for combined home and workplace ETS exposure, in hours. Results are shown for all never-smokers combined, and stratified by public ETS exposure (> 1 hour per week vs. < 1 hour per week). Those in the 4^th ^quartile of combined ETS exposure were 3 times more likely to develop RCC than those in the 1^st ^quartile (trend p = 0.020). Among never-smokers who reported public ETS exposure, those in the 4^th ^quartile of combined ETS exposure were approximately 4 times more likely to develop RCC than those in the 1^st ^quartile.

**Table 4:** Summary home/work ETS exposure (lifetime hours) among never-smokers, by public ETS exposure

	**Cases** **(%)**	**Controls** **(%)**	**OR** **(95% CI)**	**AOR** **(95% CI)**	**χ^2 ^trend^a^**
**Home/work ETS exposure, quartiles^b^**					p = 0.020
1^st ^quartile: 0 – 6,569 hours	22 (17%)	41 (31%)	1.00 (ref.)	1.00 (ref.)	
2^nd ^quartile: 6,570 – 24,454 hours	30 (23%)	38 (28%)	1.47 (0.73 – 2.98)	1.33 (0.65 – 2.72)	
3^rd ^quartile: 24,455 – 67,707 hours	34 (27%)	32 (24%)	1.98 (0.98 – 4.02)	1.92 (0.94 – 3.94)	
4^th ^quartile: 67,708 + hours	42 (33%)	23 (17%)	3.40 (1.65 – 7.03)	3.04 (1.44 – 6.42)	
Total	128 (100%)	134 (100%)			
					
**Home/work ETS exposure, quartiles – no public ETS^c^**					p = 0.214
1^st ^quartile: 0 – 6,569 hours	15 (36%)	22 (41%)	1.00 (ref.)	1.00 (ref.)	
2^nd ^quartile: 6,570 – 24,454 hours	11 (26%)	15 (28%)	1.08 (0.39 – 2.98)	1.05 (0.35 – 3.13)	
3^rd ^quartile: 24,455 – 67,707 hours	7 (17%)	12 (22%)	0.86 (0.27 – 2.68)	0.80 (0.24 – 2.68)	
4^th ^quartile: 67,708 + hours	9 (21%)	5 (9%)	2.64 (0.74 – 9.45)	2.32 (0.60 – 8.96)	
Total	42 (100%)	54 (100%)			
					
**Home/work ETS exposure, quartiles – with public ETS^c^**					p = 0.153
1^st ^quartile: 0 – 6,569 hours	7 (8%)	19 (24%)	1.00 (ref.)	1.00 (ref.)	
2^nd ^quartile: 6,570 – 24,454 hours	19 (22%)	23 (29%)	2.24 (0.78 – 6.46)	1.91 (0.62 – 5.87)	
3^rd ^quartile: 24,455 – 67,707 hours	27 (31%)	20 (25%)	3.66 (1.29 – 10.39)	3.53 (1.19 – 10.47)	
4^th ^quartile: 67,708 + hours	33 (38%)	18 (23%)	4.98 (1.76 – 14.07)	4.05 (1.35 – 12.17)	
Total	86 (100%)	80 (100%)			

^a ^Reported only for continuous variables in adjusted model

^b ^Adjusted for age, gender, race and BMI

^c ^Adjusted for age, gender, race, BMI, and weekly alcohol consumption

A stratified analysis by age was also performed to check assumptions of the previous analysis which combined all age groups. Time-dependent variables such as pack-years and years of ETS exposure are expected to increase with age. In order to assess the impact of this co-linearity, descriptive statistics for time-dependent variables were calculated, and models were fit, for separate age groups (Tables 5 and 6). For all variables, those subjects younger than 50 years old were consistently below mean exposure values. Furthermore, only subjects between 50 and 80 years old had adequate observations of 20 or more pack-years, and interpretation of pack-years associations reported in this paper should therefore be limited to that age group. With smoking variables and age both treated as continuous, the correlation coefficient was of notable magnitude only for smoking cessation (Spearman's *ρ *= 0.388, p < 0.0001). Although this suggests potential confounding by age, the expected effect of confounding is contrary to the observed trend. Risk of RCC is known to increase with age, which would produce a trend of increasing risk (or moderated protective effect) as years of cessation increase. The present findings show that risk clearly decreases with increasing years of smoking cessation. Descriptive statistics for years of home and workplace ETS exposure across age groups show little evidence of notable co-linearity.

**Table 5:** Descriptive statistics for cigarette smoking and ETS exposure (home and workplace), by age-decade^a,b^

**Age (years)**	**Cigarette duration (yrs.)^c^**		**Cigarette pack-years^d^**		**Cigarette cessation (yrs.)^e^**		**Home ETS years^f^**		**Work ETS years^g^**	
	Mean	St. dev.	Mean	St. dev.	Mean	St. dev.	Mean	St. dev.	Mean	St. dev.
**20 – 29**	2.80	3.36	0.61	0.92	4.00	-	2.50	3.00	1.00	2.24
**30 – 39**	7.62	8.95	4.18	5.75	4.33	4.16	11.14	9.87	2.29	3.73
**40 – 49**	11.30	13.75	8.36	13.36	14.29	11.18	13.93	11.39	7.16	9.76
**50 – 59**	17.05	16.06	18.09	22.33	16.85	12.05	13.78	13.99	8.11	10.84
**60 – 69**	20.64	19.76	20.68	27.45	23.20	13.34	17.00	16.43	12.88	14.53
**70 – 79**	19.72	20.45	21.08	27.74	28.61	15.50	19.00	16.30	10.28	14.39
**≥ 80**	12.88	18.13	11.69	25.56	36.48	16.16	19.03	21.17	9.26	15.49

**Total**	17.44	18.67	17.49	25.33	24.66	15.39	16.45	16.17	9.65	13.40

^a ^Spearman's correlation coefficients reported for both variables as continuous.

^b ^Cigarette duration and pack-years reported for full sample (N = 672); Cigarette cessation reported for ex-smokers only (N = 305); ETS statistics reported for never-smokers only (N = 263).

^c ^F = 5.163, p < 0.0001; Spearman's *ρ *= 0.048, p = 0.215

^d ^F = 4.668, p < 0.0001; Spearman's *ρ *= 0.022, p = 0.564

^e ^F = 10.822, p < 0.0001; Spearman's *ρ *= 0.388, p < 0.0001

^f ^F = 1.354, p = 0.234; Spearman's *ρ *= 0.102, p = 0.121

^g ^F = 1.699, p = 0.122; Spearman's *ρ *= 0.029, p = 0.636

**Table 6:** Adjusted odds ratios for renal cell carcinoma by age-decade: Cigarette smoking (pack-years) and home ETS (years)

	**Cigarette smoking (pack-years)^a^**			**Home ETS (years)^b^**		
	**Cases (%)**	**Controls (%)**	**AOR** **(95% CI)^c^**	**Cases (%)**	**Controls (%)**	**AOR** **(95% CI)^c^**

**40 – 49 years**						
Reference	14 (56%)	18 (47%)	1.00	5 (36%)	5 (28%)	1.00
Level 1	6 (24%)	14 (37%)	0.48 (0.13 – 1.70)	6 (43%)	8 (44%)	0.85 (0.15 – 5.01)
Level 2	5 (20%)	6 (16%)	1.26 (0.29 – 5.55)	3 (21%)	5 (28%)	0.83 (0.11 – 6.27)
Total	25 (100%)	38 (100%)		14 (100%)	18 (100%)	
						
**50 – 59 years**						
Reference	22 (35%)	23 (36%)	1.00	5 (23%)	7 (30%)	1.00
Level 1	12 (19%)	21 (33%)	0.69 (0.26 – 1.78)	10 (46%)	13 (57%)	0.66 (0.14 – 3.11)
Level 2	29 (46%)	20 (31%)	1.61 (0.69 – 3.79)	7 (32%)	3 (13%)	2.20 (0.30 – 15.94)
Total	63 (100%)	64 (100%)		22 (100%)	23 (100%)	
						
**60 – 69 years**						
Reference	40 (37%)	29 (30%)	1.00	13 (33%)	7 (25%)	1.00
Level 1	26 (24%)	33 (34%)	0.57 (0.28 – 1.15)	10 (25%)	14 (50%)	0.35 (0.08 – 1.49)
Level 2	42 (39%)	36 (37%)	0.84 (0.43 – 1.64)	17 (43%)	7 (25%)	1.17 (0.28 – 4.94)
Total	108 (100%)	98 (100%)		40 (100%)	28 (100%)	
						
**70 – 79 years**						
Reference	32 (34%)	32 (40%)	1.00	6 (19%)	12 (38%)	1.00
Level 1	23 (24%)	21 (26%)	1.06 (0.49 – 2.31)	6 (19%)	11 (34%)	1.17 (0.28 – 4.97)
Level 2	40 (42%)	28 (35%)	1.32 (0.64 – 2.71)	20 (63%)	9 (28%)	6.29 (1.50 – 26.29)
Total	95 (100%)	81 (100%)		32 (100%)	32 (100%)	
						
**≥ 80 years**						
Reference	21 (51%)	22 (61%)	1.00	6 (30%)	7 (32%)	1.00
Level 1	9 (22%)	9 (25%)	0.90 (0.26 – 3.12)	6 (30%)	7 (32%)	1.48 (0.24 – 9.14)
Level 2	11 (27%)	5 (14%)	2.18 (0.62 – 7.70)	8 (40%)	8 (36%)	1.43 (0.30 – 6.86)
Total	41 (100%)	36 (100%)		20 (100%)	22 (100%)	

^a ^Cigarette smoking

Reference: never-smoker

Level 1: ≤ 20 pack-years

Level 2: > 20 pack-years

^b ^Home ETS

Reference: never exposed

Level 1: ≤ 20 years

Level 2: > 20 years

^c ^Adjusted for gender, race, and BMI

Table 6 presents stratified analyses of cigarette smoking (in pack-years) and home ETS (in years) at ten-year age intervals (Table 6). Results for subjects younger than 40 years old were excluded from this table because low sample size within these age strata precluded meaningful results. Within each age group, risk of RCC increased consistently with increasing levels of pack-years and years of home ETS. Associations between RCC and home ETS were particularly strong in the 70- to 79-year age group, with greater than 20 years of exposure increasing risk by a magnitude of six, compared with no exposure.

## Discussion

This study observed a 30% RCC risk increase among smokers of 20 or more pack-years compared with never-smokers. This finding is consistent with those of previous case-control studies [7,15-19], and conflicts with only two [20,21] for which pack-years were reported. Cohort studies have also reported positive associations between smoking and kidney cancer incidence [22,23] or mortality [24,25]. For smokers of 20 or more pack-years, we observed a more significant risk increase when the reference group included both never-smokers and those smoking less than 20 pack-years. The apparent protective effect of light smoking is likely explained by the high proportion of ex-smokers (90%) among those classified as light smokers.

While the etiologic link between smoking and RCC is well established, these results contribute to existing knowledge using a refined measure that accounts for variations in lifetime smoking patterns. Retrospective methods for calculating pack-years that take into account temporary periods of cessation have been found moderately valid when compared against prospective methods [14]. This study modifies this method further by assessing smoking patterns across discrete age intervals. Correspondence of these estimates with those found in the literature lends weight to the potential validity of home ETS measures used in this study, which relied on the same measurement principles. However, it should be noted that our methods for assessing pack-years require further study for validation.

The protective effects of smoking cessation were particularly strong in this study. These findings are more conclusive than those of other studies evaluating RCC risk among ex-smokers [6,15-20,26,27], although differing reference categories preclude direct comparisons. Yuan et al. (1998) found that, compared with current smokers, those who had quit smoking for 10 or more years experienced a 30% RCC risk reduction [28]. Parker et al. (2003) reported a 50% reduction in risk among those who had quit for 30 or more years, compared with current smokers [27]. Our analysis was performed among ex-smokers only, using as a reference those who had quit 1–10 years prior to interview. This study had an average follow-up for cases of three years, and it is likely that many cases who were current smokers prior to diagnosis would have quit by the time of interview. Analyses of cessation using current smokers as a reference would therefore have resulted in an artificially lower proportion of cases in the reference group – a potential bias that could underestimate the protective effect of cessation.

While this study found increased RCC risks among ever-smokers who reported inhaling, these associations were attenuated after controlling for pack-years. Mellemgaard (1995) reported a similar confounding effect, in which the association between inhalation and risk was likely explained by the fact that heavier smokers tended to inhale more frequently [17]. Other studies have reported conflicting results on inhalation and RCC [6,16,28], particularly when inhalation patterns were evaluated across "deep", "moderate" and "light" categories. A dichotomous inhalation measure is probably sensitive enough to detect potential effects.

This study is among the first to observe associations between RCC and home ETS exposure among never-smokers. We know of only two other case-control studies to date that have evaluated associations between ETS and RCC [6,7]. Both studies assessed self-reported home and workplace ETS exposure in Canadian populations using mailed questionnaires. Krieger et al. (1993) observed non-significant risk increases for both non-smoking men (OR = 1.6) and women (OR = 1.7) reporting greater than 8 hours of daily ETS exposure [6]. Hu et al. (2005) reported significant risk increases for 43 or more years of combined home and workplace ETS exposure (compared with those never exposed) among non-smoking men (OR = 3.9) and women (OR = 1.8) [7]. The present study reports a comparable OR of 3.04 for the highest quartile of combined home and workplace ETS exposure.

The implausibility of ETS risk ratios that exceed risk ratios for direct smoking – both in this study and in the literature – may be partly explained by the differing composition of reference groups. Analyses of smoking are performed for the entire sample with never-smokers as the reference group, including those exposed to ETS – an effect that may slightly increase the odds of being a case in the reference group (from Table 2, odds: 131/134 = 0.98) and thus dampen the estimated odds ratio. Conversely, analyses of ETS exposure are performed on the subsample of never-smokers, among whom the reference group has no history of ETS exposure and lower odds of being a case (from Table 5, odds: 35/43 = 0.81). In Table 2, substitution of the lower odds for the "purer" referent group (neither direct nor indirect smoke exposure) into the calculation of an unadjusted OR for smoking greater than 20 years would increase the OR from 1.33 to 1.60. From Table 3, the comparable unadjusted OR for home ETS exposure of greater than 20 years is 2.15. Thus, home ETS still appears to have a larger effect than direct smoking, even after using the same reference group. A basic statistical explanation for this is that the ETS effect estimates are based on much smaller samples and are highly variable; the true ETS effects are probably smaller than reported here. A fully satisfactory explanation for why ETS appears to have a larger effect than direct smoking is lacking; a reasonable hypothesis for future research is that the differences in effect estimates reflect the as-yet unclear role of confounders such as dietary and other lifestyle factors.

Home ETS was assessed by both years of exposure, which does not account for intensity, and lifetime hours of exposure – a computed variable that incorporates both duration and intensity – resulting in different standards of precision. The risk increases reported here are based on variables assessed by interviewers trained in retrospective data collection – a method that typically provides higher estimates of sensitivity and specificity [29]. However, unlike ETS measurement based on shorter recall periods [8,30], methods for measuring long-term, retrospective ETS exposure have not been validated.

While this study reported associations between RCC and home ETS, it remains unclear why similar associations were not found for workplace or public ETS. It is possible that potential associations were not detected due to measurement error. While ETS exposure in the home was measured using separate estimates for each home in which the subject lived with a smoker, workplace ETS was collected as an overall estimate, and only frequency data were collected for public ETS. Self-reported ETS measures in the workplace may be problematic due to variations in room volume, ventilation, and employee turnover [8,31]. Furthermore, concentrations of both workplace and public ETS vary by setting, with higher concentrations in indoor locations and those with a greater number of smokers (e.g., bars and restaurants). The public ETS measure for the present study did not differentiate between exposure in cars or public transportation (high concentration) and exposure in outdoor locations, such as bus stops or restaurant patios (low concentration), resulting in low specificity and possibly contributing to exposure misclassification.

Although these results suggest that home ETS is implicated in the etiology of RCC, a number of limitations must be acknowledged. The low response rate among controls (42%) suggests that selection biases may have been present, with controls choosing to participate having healthier lifestyle patterns than those who refuse. This potential bias would result in lower exposure rates within the control sample compared to the general population, producing overestimated measures of association [12]. In a study of ETS exposures among female teachers in California, the prevalence of lifetime ETS exposure in the home ranged from 45% to 78%, depending on the teacher's birth decade [32]. The highest prevalence was observed among teachers born in the 1930s (78%). In comparison, among controls in the present study aged 60 – 69 years or 70 – 79 years, lifetime home ETS prevalence was 75% and 62%, respectively. The home ETS estimates collected from the control sample may therefore be slightly lower than that found in the general population, suggesting that their corresponding odds ratios (e.g., 2.18 for greater than 20 years of exposure) overestimate the true measure of association. While the magnitude of this study's home ETS estimates may be in question, one may still conclude from these findings that long-term ETS exposure in the home is a likely risk factor for RCC. Low response among controls is a significant limitation in the present study, but it should be noted that our response rate reflects an observed trend in epidemiologic studies over the past thirty years [12,33,34], with participation rates for population-based case-control studies declining nearly 2% per year [33].

The age discrepancy between cases and controls occurred from obtaining initial matching frequencies from SEER data, which are based on cancer incidence reporting at the national level. Because lifetime smoking and ETS variables are time-dependent, the potential of co-linearity or confounding with age was a concern. Univariate descriptive statistics on selected tobacco variables showed expected increases in exposure levels with age, but little evidence of confounding (with the exception of smoking cessation) (Tables 5 and 6). Trends in the association between RCC and increasing levels of pack-years or years of home ETS did not differ substantially with age (Table 6).

Among cases, the average follow-up was three years, leading to potential survivor bias in which excluded deceased cases may have had different exposure rates than those included in the sample. However, with regard to smoking, higher rates of exposure are more likely among deceased cases, making findings more conservative. This follow-up duration also introduces the potential for recall bias, with cases interviewed shortly after diagnosis having more accurate recall than those interviewed distant to diagnosis. Estimates calculated using a subset of 92 cases interviewed within two years of diagnosis were not appreciably different from those calculated using the full case sample, suggesting that inclusion of cases with longer follow-up did not contribute to differential recall (results not shown).

Lastly, the cases in this study represent all histologic subtypes of RCC, of which clear cell RCC is the most common (85% to 90% of all renal tumors) [35]. Research has shown that the different RCC subtypes produce varied prognosis and outcomes in patients, and are morphologically and genetically distinct [36]. It is likely that the RCC subtypes are also etiologically distinct, and inclusion of all subtypes in a case-control study may affect the validity of measures of association for the risk factors in question.

## Conclusion

The findings from this study correspond with those of prior studies that have reported associations between cigarette smoking and RCC and protective effects for smoking cessation. Our results show that ETS exposure in the home may act as an independent risk factor for RCC. While the association is biologically plausible, additional research is needed to validate these findings. More sensitive self-report measures of ETS exposure are needed which assess exposure by location, duration, and intensity.

## Abbreviations

RCC: renal cell cancer; ETS: environmental tobacco smoke; OR: odds ratio; CI: confidence interval; RDD: random-digit dialing; SEER: Surveillance, Epidemiology and End Results; BMI: body mass index

## Competing interests

The authors declare that they have no competing interests.

## Authors' contributions

RPT participated in coordination of the study, collection of data, statistical analysis, and drafted the manuscript. SMDG participated in collection of data, assessment of dietary factors, and review of the manuscript. DB conducted and guided statistical analysis for the study and reviewed the manuscript. TS provided medical consulting for all aspects of the study. NRA conceived of the study and participated in its design and coordination.

## Pre-publication history

The pre-publication history for this paper can be accessed here:


